# Feed-additive probiotics accelerate yet antibiotics delay intestinal microbiota maturation in broiler chicken

**DOI:** 10.1186/s40168-017-0315-1

**Published:** 2017-08-03

**Authors:** Pengfei Gao, Chen Ma, Zheng Sun, Lifeng Wang, Shi Huang, Xiaoquan Su, Jian Xu, Heping Zhang

**Affiliations:** 1 Key Laboratory of Dairy Biotechnology and Engineering, Inner Mongolia Agricultural University, Hohhot, 010018 China; 2 Single-Cell Center, CAS Key Laboratory of Biofuels and Shandong Key Laboratory of Energy Genetics, Qingdao Institute of BioEnergy and Bioprocess Technology, Chinese Academy of Sciences, Qingdao, Shandong 266101 China

**Keywords:** Probiotics, Intestinal microbiota, Broiler, Antibiotic overuse, Antibiotic resistance

## Abstract

**Background:**

Reducing antibiotics overuse in animal agriculture is one key in combat against the spread of antibiotic resistance. Probiotics are a potential replacement of antibiotics in animal feed; however, it is not clear whether and how probiotics and antibiotics differ in impact on physiology and microbial ecology of host animals.

**Results:**

Host phenotype and fecal microbiota of broilers with either antibiotics or probiotics as feed additive were simultaneously sampled at four time points from birth to slaughter and then compared. Probiotic feeding resulted in a lower feed conversion ratio (FCR) and induced the highest level of immunity response, suggesting greater economic benefits in broiler farming. Probiotic use but not antibiotic use recapitulated the characteristics of age-dependent development of gut microbiota in the control group. The maturation of intestinal microbiota was greatly accelerated by probiotic feeding, yet significantly retarded and eventually delayed by antibiotic feeding. LP-8 stimulated the growth of many intestinal *Lactobacillus* spp. and led to an altered bacterial correlation network where *Lactobacillus* spp. are negatively correlated with 14 genera and positively linked with none, yet from the start antibiotic feeding featured a less-organized network where such inter-genera interactions were fewer and weaker. Consistently, microbiota-encoded functions as revealed by metagenome sequencing were highly distinct between the two groups. Thus, “intestinal microbiota maturation index” was proposed to quantitatively compare impact of feed additives on animal microecology.

**Conclusions:**

Our results reveal a tremendous potential of probiotics as antibiotics’ substitute in poultry farming.

**Electronic supplementary material:**

The online version of this article (doi:10.1186/s40168-017-0315-1) contains supplementary material, which is available to authorized users.

## Background

Antibiotic intake of food animals, as well as the resulted antibiotic residue in food, has been recognized as one leading cause of the rapid spread of antimicrobial resistance in human populations [1, 2]. Abusive feeding of antibiotics to food animals causes the direct selection for antibiotic-resistant microbes and turns the food animal systems into reservoirs of antibiotic resistance genes. Moreover, antibiotic intake of human via inadvertent consumption of such antibiotics-contaminated food can undermine efficacy of antibiotics in combating bacterial infections, hinder normal development of gut microbiota, and eventually increase risk of chronic diseases [3, 4]. The scope of antibiotic residue contamination in food animals is alarming: a recent study of over 1000 8~11-year-old children in Shanghai, China, detected in 58% of the urine samples multiple antibiotics that are only used in food animals (i.e., tylosin, chlortetracycline, and enrofloxacin) [5]. Thus, reducing antibiotic intake and eliminating antibiotic residues in food animal agriculture has become one priority in food safety and public health.

Antibiotic residues in food animals is a consequence of antibiotic overuse in animal feed [6]. With over 50 billion animals reared annually as human food source for both meat and eggs, chicken are the most common type of poultry and contribute one third of meat production worldwide. However, 20~50% fresh or frozen broilers were antibiotic-residue positive, due to the administration of antibiotics in broiler feed [7–10]. In chicken farming, efforts seeking alternatives for in-feed antibiotics started in the 1980s and have gained enormous interest in recent years [11]. Such alternatives include fiber-degrading enzymes, prebiotics, probiotics, symbiotics, and phytobiotics [12]. Among them, probiotics are advantageous for its low production cost and wide range of application among different kinds of host animals [13]. For example, a meta-analysis of 35 studies of probiotics across Brazil between 1995 and 2005 indicated that probiotics are technically viable alternatives to antibiotics in broiler chicken feed [14].

As the first officially issued feed-additive microorganisms, *Lactobacillus* spp. are considered an advanced alternative to antibiotics and have been used in feed processing for decades due to their beneficial effects on immunity, metabolism, and growth of livestock [15, 16]. For example, *Lactobacillus plantarum* strain 8 (i.e., “LP-8”), originally isolated from naturally fermented yoghurt of a herdsmen family in the Wulatezhongqi grassland of Inner Mongolia, China, is able to survive and multiply in human intestinal tract [17]. Administration of LP-8 in human adults conveys antagonistic properties against pathogenic bacteria, increases the content of intestinal mucosal immune globulin A (SIgA), and enhances the antioxidant capacity [18]. Moreover, in broiler chicken, LP-8 was shown to improve growth performance, nutrient digestibility, immunity, and intestinal health [19], suggesting the potential value of LP-8 as an alternative to antibiotics in chicken farming. However, it is not clear whether and how the probiotics and the antibiotics differ in impact on physiology and microbial ecology of animal hosts [20].

Here by simultaneously tracking host phenotypes and fecal microbiome structure of broilers along the full span from birth to slaughter, we compared the development of host physiology and intestinal microbiota among the two feed additives and a normal diet control. The results revealed that probiotic feeding resulted in a lower feed conversion ratio (FCR) and induced the highest level of immune response. Moreover, probiotics but not antibiotics recapitulated the characteristics of age-dependent development of gut microbiota in the control group. Furthermore, maturation of intestinal microbiota was greatly accelerated by probiotic feeding, yet significantly retarded and eventually delayed by antibiotic feeding. Consistently, profound functional distinction in intestinal microbiota was revealed by metagenomic sequencing between the probiotic and the antibiotic groups. Co-occurrence network analysis revealed LP-8 supplementation stimulated the abundance of many intestinal *Lactobacillus* spp. and led to a tightly organized bacterial correlation network where *Lactobacillus* spp. are negatively correlated with 14 bacterial genera and positively linked with none; in contrast, from the start, antibiotic feeding featured a much less organized network, indicating disruption of numerous inter-genera interactions. Thus, LP-8 feeding accelerated maturation of intestinal microbiota by promoting growth of the indigenous *Lactobacillus* spp. in broiler intestine and then together inhibiting other intestinal genera. These results revealed remarkable distinction between probiotics and antibiotics in their impact on broiler microbiota development, and underscore the tremendous potential of probiotics as antibiotics’ substitute in poultry farming.

## Results

### Development of broiler intestinal microbiota with either probiotics or antibiotics as feed supplement

A total of 270 1-day-old Cobb 500 broilers were first randomly divided into three groups: they were either fed a base diet (i.e., the control group), the base diet plus the antibiotics of chlortetracycline and salinomycin at 500 g/ton-of-feed each (the antibiotic group), or the base diet plus LP-8 in drinking water (the probiotic group; “Methods”). Every group (i.e., 90 broilers) was then equally divided into six pens randomly. Each such 15-broiler pen thus served as a biological replicate and was tracked for physiological, immunological, and intestinal microbiome structure for 42 days (i.e., from birth to slaughter). To evaluate the performance in growth promotion, average daily gain (ADG; per broiler), average daily feed intake (ADFI; per pen), and FCR (per pen) were recorded. To assess the immunological response, immune indices including immune organ indices, serum IgG, and intestinal secretory IgA were measured for specific organs and tissues [21]. To probe the development of intestinal microbiota, 16S ribosomal RNA (rRNA) amplicon sequencing was performed for feces collected on day 7, 28, and 42 from 12 randomly picked broilers from each group, plus those from 12 randomly picked broilers on day 0 (i.e., before any treatments; “Methods”). Furthermore, 10 fecal samples were collected from each of the three groups on day 42 for total metagenome sequencing (“Methods”), for functional comparison of the intestinal microbiota.

### Probiotics and antibiotics both conveyed growth benefits yet only probiotics activated protective host immune responses

**Fig. 1:**
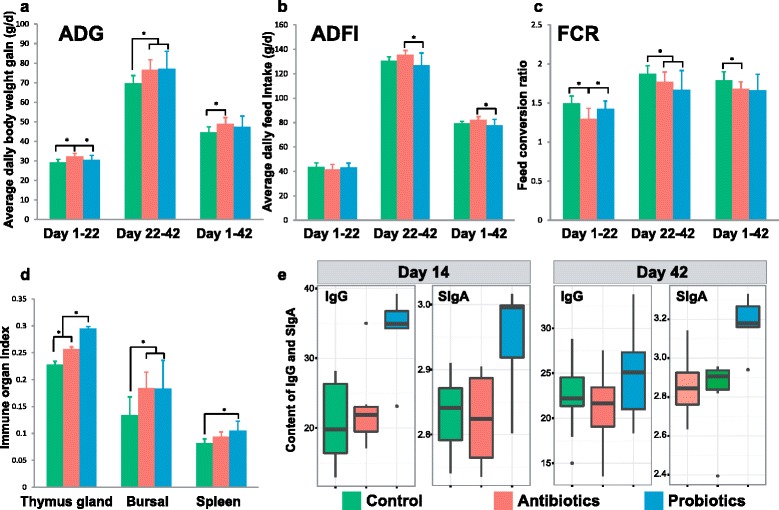
Growth performance and immune activity of broiler chicken under the feed-additive regimens of antibiotics, probiotics, and the control. Average daily gain (ADG; *n* = 6; **a**), average daily feed intake (ADFI; *n* = 6; **b**), and feed conversion ratio (FCR; *n* = 6; **c**) were compared among the three treatment groups during the various periods. “*”: significantly changed (*p* < 0.05). The average daily gain (ADG) was measured per chicken, while the average daily feed intake (ADFI) and the feed conversion ratio (FCR) were measured per 15-broiler pen (six pens in each group). **d** Comparison of three immune organ indexes from thymus gland, bursa, and spleen (*n* = 6), which were measured at the last day of the 42-day regiment. Two chickens were randomly selected from each replicate of each group and sacrificed for the measurements. **e** Comparison of serum IgG (*n* = 6) and intestinal SIgA (*n* = 6) levels among the three groups on day 14 and day 42. The two values were measured on a per chicken basis, and 12 of the chickens from each pen were randomly selected for the inter-group comparison

All chickens were healthy throughout the feeding trial period. During the first 22 days, no significant ADFI change (*P* > 0.05) was detected among the three groups, but the antibiotic group exhibit 10.6 and 5.9% higher ADG than control and probiotic group, as for FCR, the antibiotic group is 13.4 and 9.1% lower than the control and probiotic group (Fig. 1a–c). In the next 21 days, both the antibiotic and the probiotic groups produced a 9.8 and 10.4% higher ADG than the control; however, the probiotic group consumed less food and consequently exhibit a 5.9% lower FCR than the antibiotic group. Over the complete birth-to-shelf process of 42 days, probiotic feeding produced a level of weight gain (i.e., ADG) that is identical to antibiotic feeding (both 10.3 and 6.7% higher than the control) and a significantly lower FCR, suggesting that P-8 provided equivalent or greater benefits in weight gain, feed intake, and feed efficiency as antibiotics did (Additional file 1: Table S1).

Induction and maintenance of an appropriate level of immunological activity is crucial for healthy broiler growth in poultry farms [22]. For the broilers, a number of key immunological indices were compared among the regimens of feed additives. *Firstly*, immune organ indices, referred to as immune organ weights and commonly used in poultry industry as a measurement for immunity [23, 24], were measured for each of the thymus gland, bursa, and spleen (“Methods”). Thymus is a central immune organ that plays an important role in inducing T lymphocytes differentiation and maturation, while bursa is a bird-specific humoral immune organ. Spleen, as the biggest peripheral immune organ, is involved in immune reaction of chicken. The immune organ indices of the thymus gland, bursa, and spleen on day 42 were 29.3, 36.5, and 28.0% higher in the probiotic group than the control, and immune organ index of the thymus gland was 14.7% higher in the probiotic group than the antibiotic group, indicating a most enhanced immunity in the probiotic group (Fig. 1d).

*Secondly*, serum IgG and intestinal secretory IgA were compared among the groups (“Methods”), as serum IgG reflects the system immune state, while intestinal secretory IgA reflects the intestinal immunity state [25]. On day 14, the probiotic group exhibit 63.7 and 48.0% higher expression level of serum IgG respectively than the control and the antibiotic groups and, moreover, 4.2 and 4.6% higher intestinal secretory IgA than the other two groups. On day 42, the probiotic group exhibited 19.5% higher expression level of serum IgG than the control and moreover 11.2 and 12.4% higher intestinal secretory IgA respectively than the other two groups (Fig. 1e). The highest level of IgG and IgA expression as detected in the probiotic group indicated a boosted immunity after probiotic feeding.

### Oral administration of LP-8 elevated relative abundance of a wide range of indigenous *Lactobacillus* species in intestinal microbiota

*Lactobacillus* spp. are widely considered as beneficial to both humans and animals, thus high content of *Lactobacillus* spp. is linked to the wellbeing of chicken [26]. For example, *L. paracasei* reportedly enhances the phagocytic activity of the gut cells of poultry (including chicken [27]) and *L. plantarum* also exerted strong stimulation effect on chicken gut cells [28].

**Fig. 2:**
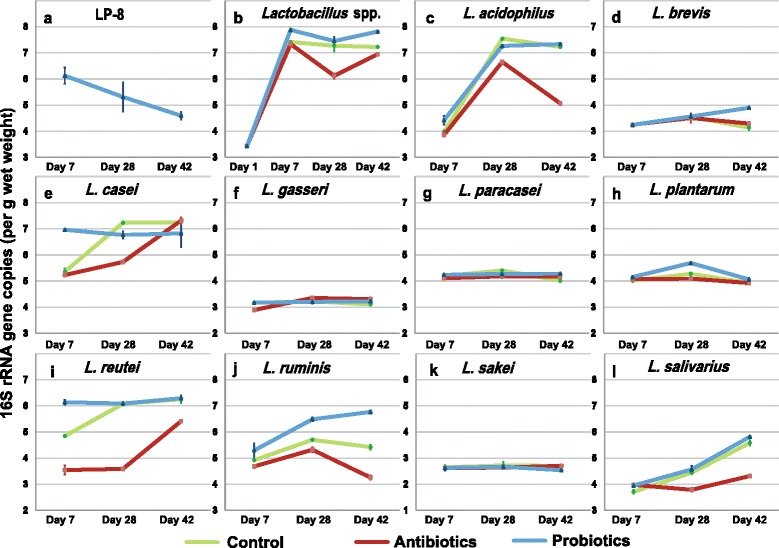
Oral administration of LP-8 elevated relative abundance of a wide range of indigenous *Lactobacillus* species in intestinal microbiota. In the fecal samples, abundance of LP-8 (**a**), total *Lactobacillus* spp. (**b**), as well as the intestinal *Lactobacillus* spp. of *L. acidophilus* (**c**), *L. brevis* (**d**), *L. casei* (**e**), *L. gasseri* (**f**), *L. paracasei* (**g**), *L. plantarum* (**h**), *L. reutei* (**i**), *L. rumins* (**j**), *L. sakei* (**k**), and *L. salivarius* (**l**) were determined by quantitative PCR on day 7, day 28, and day 42 for each of the three regimens

To test the ability of *L. plantarum* strain LP-8 to access the gut and the impact of LP-8 feeding on the intestinal *Lactobacillus* species, abundance of LP-8 and nine other *Lactobacillus* species from fecal samples were compared on day 7, 28, and 42 among the groups using RT-PCR (Additional file 2: Table S6), which is able to distinguish microbiota at the strain level. In the probiotic group, LP-8 reached 6.03 ± 0.18 Log10CFU/g on day 7, was reduced to 4.84 ± 0.10 Log10CFU/g on day 28 and then 4.67 ± 0.09 Log10CFU/g on day 42 (Fig. 2a). LP-8 was not detected in the other two groups. Thus, throughout the feeding period, LP-8 has survived in the digestive system and reached the broiler intestine.

Interestingly, oral administration of LP-8 resulted in remarkable enrichment of non-LP-8 *Lactobacillus* spp. in intestinal microbiota. In the probiotic group, on day 7, 28, and 42, nine species of *Lactobacillus* beyond LP-8 that included *L. acidophilus, L. brevis*, *L. casei*, *L. gasseri*, *L. paracasei*, *L. plantarum*, *L. reutei*, *L. ruminis*, *L. sakei*, and *L. salivarius* were detected. By day 7, in the probiotic group, *L. acidophilus*, *L. casei*, *L. paracasei*, *L. plantarum*, *L. reutei*, *L. ruminis*, *and L. salivarius* were all significantly elevated (by 11.1, 29.8, 1.1, 3.4, 26.6, 7.6, and 6.4% respectively), yet abundance of *L. brevis*, *L. gasseri*, and *L. sakei* did not respond to LP-8 supplementation. In the antibiotic group, however, *L. acidophilus*, *L. casei*, *L. gasseri*, *L. paracasei*, *L. reutei*, and *L. ruminis* all decreased (by 2.9, 2.4, 8.7, 1.9, 26.8, and 4.9% respectively), although *L. plantarum* and *L. salivarius* slightly increased (by 1.4 and 7.1%; Additional file 3: Fig. S1a). At day 28, in the probiotic group, the levels of *L. plantarum* (9.6%), *L. ruminis* (13.7%), and *L. salivarius* (2.7%) are higher than those in the control group, while those of *L. acidophilus* (3.7%), *L. casei* (6.4%), and *L. paracasei* (3.0%) are lower than those in the control; however, in the antibiotic group, all the Lactobacillus strains were reduced as compared to those in the control (except that *L. gasseri* increased by 4.0%; Additional file 3: Fig. S1b). At day 42, probiotic intake elevated the abundance of *L. acidophilus* (by 1.4%), *L. brevis* (by 24.1%), *L. gasseri* (by 3.8%), *L. paracasei* (by 6.6%), *L. plantarum* (by 3.4%), *L. ruminis* (by 24.7%), and *L. salivarius* (by 4.1%), whereas *L. casei* and *L. sakei* reduced by 5.9 and 6.0%; on the other hand, antibiotic intake resulted in the reduction of *L. acidophilus* (by 29.9%), *L. reutei* (by 13.4%), *L. ruminis* (by 21.4%), and *L. salivarius* (by 22.7%), as well as the elevation of *L. brevis* (by 4.9%), *L. gasseri* (by 6.4%), and *L. paracasei* (by 3.9%; Additional file 3: Fig. S1c).

Therefore, over the full course of 42 days, *Lactobacillus* spp. abundance was the highest in the probiotic group while the lowest in the antibiotic group (Fig. 2b). Moreover, based on their antibiotic/probiotic sensitivity, the nine *Lactobacillus* spp. can be grouped into (*i*) the insensitive cluster, including *L. gasseri*, *L. paracasei*, and *L. sakei* (Fig. 2f–k), (*ii*) the slightly sensitive cluster, including *L. brevis* and *L. plantarum* which differed with those in the control group only at selected time points (Fig. 2d, h), and (*iii*) the highly sensitive cluster, including *L. acidophilus*, *L. casei*, *L. reutei*, *L. ruminis*, and *L. salivarius*, which mostly were inhibited by antibiotics yet stimulated by probiotics (Fig. 2c–l).

### LP-8 accelerated, yet antibiotics delayed, the maturation process of broiler intestinal microbiota

**Fig. 3:**
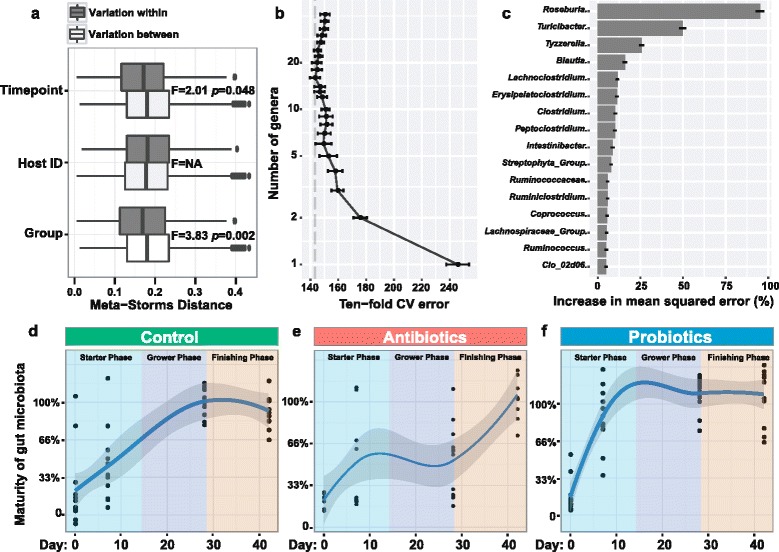
Comparison of intestinal microbiota maturation among the three regimens. **a** Contributions of different factors on chicken gut microbiota variation. The PERMANOVA test was used for all samples based on Meta-Storm distance. Group is the largest contributor (*F =* 3.83, *p* = 0.002) for broiler gut microbiota variation followed by time point (*F =* 2.01, *p* = 0.048). **b** Tenfold cross-validation error as a function of the number of input genera-level taxa used to regress against the age of chicken in the training set, in the order of variable importance. **c** The top 16 age-discriminatory bacterial taxa as identified via the Random Forest regression. These taxa were considered as bacterial biomarkers that are able to differentiate the maturity of broiler intestinal microbiota from birth to slaughter. The temporal patterns of intestinal maturation were compared among the control group (**d**), the antibiotic group (**e**), and the probiotic group (**f**), which revealed that probiotics accelerated yet antibiotics delayed intestinal microbiota maturation

Administration of LP-8 and antibiotics also induced a significant change to broiler intestinal microbiota. PERMANOVA test based on Meta-Storm distance revealed that both time point and feed additive have a significant effect on the fecal microbiome structure (“Methods”, Additional file 4: Table S2). Feed additive (LP-8 or antibiotics) is the most important contributor of microbiota variation (*F* = 3.83, *p* = 0.002), as difference between LP-8 and antibiotics is consistently larger than the time point (i.e., age; *F* = 2.01, *p* = 0.048) or the variation among animal individuals (Fig. 3a). Thus, pinpointing the discriminating microbial features among feed additives would first require identification of the age-dependent microbiota features.

To probe the age-dependent development of broiler gut microbiota, age-discriminatory taxa were identified by respectively regressing the relative abundance of the entire list of genera against the corresponding chronologic age of chicken in the control group (“Methods”). In this way, 29 age-discriminatory taxa were identified. Among them, a short list of top genera were used for the subsequent construction of the microbiota-based model for discriminating different developmental stages, i.e., degree of microbiota maturity, as inclusion of any taxa beyond these top taxa produced only minimal improvement in model performance (Fig. 3b). This model which consists of 16 genera is able to distinguish the maturity of intestinal microbiota during the 42 days (56.68% variation explained; Fig. 3c).

To probe the effect of feed additive on microbiota maturation, development of microbiota in the probiotic and the antibiotic groups as defined by the age-discriminatory taxa identified above were monitored. Specifically, the Random Forest model was trained on the control group to identify age-discriminant taxa and then modeling of the microbiota age was performed on those same taxa across all three groups. Intriguingly, the patterns of microbiota development were highly distinct. The natural development of microbiota (i.e., in the control group when neither antibiotics nor probiotics were supplemented) exhibited a smooth curve that gradually grows until reaching plateau at day 30 (Fig. 3d). However, the curve in the antibiotic group featured a late-maturing pattern that does not reach the plateau until day 40, suggesting a delay of approximately 10 days in microbiota development as compared to the control group (Fig. 3e). In contrast, in the probiotic group, the curve exhibited an early-maturing pattern, which reaches plateau in as early as day 15, indicating an acceleration of intestinal microbiota maturation by approximately 15 days (Fig. 3f). The apparent early maturation of intestinal microbiota is consistent with the early development of immunity in the probiotic group (Fig. 2b). Thus apparently, probiotic and antibiotic administrations generated opposite effects on the age-dependent maturation of intestinal microbiota, with the former accelerating the process whereas the latter delaying it (the relative abundance change of the 16 age-discriminatory taxa are shown in Additional file 5: Fig. S2). In addition, from day 1 to day 42, the beta diversity of intestinal microbiota changed more heavily in the antibiotic group (*F* = 0.164, *p* = 0.003; ANOSIM) than in either the control group (*F* = 0.136, *p* = 0.003) or the probiotic group (*F* = 0.149, *p* = 0.003).

To quantitatively define the speed of microbiota maturity and thus compare the impact of feed additives (and diet in general) on microbiota development, we propose an index called “intestinal microbiota maturation index” (IMMI), which is defined as “time required to reach the full maturity of gut microbiota as defined by the additive-free group” (“Methods”). Interestingly, for the control group, the developmental pattern of broiler intestinal microbiota revealed that the timing of microbiota reaching the plateau (i.e., “full maturity”) actually coincided with the start of the finishing phase, i.e., when the chicken start to rapidly gain body weight (Fig. 3d). This suggests a link between intestinal microbiota and growth performance in broiler farming. On the other hand, administration of LP-8 and that of the antibiotics carry a IMMI of 15 and 40 respectively, as compared to a IMMI of 30 for the control group.

### Organismal features of intestinal microbiota in the probiotic and the antibiotic treatments

**Fig. 4:**
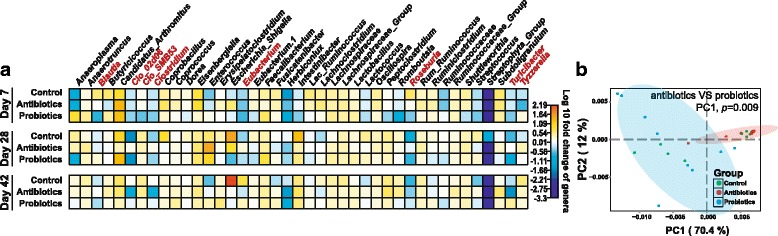
Patterns of organismal and functional-gene abundance for the broiler intestinal microbiota in the three regimens. **a** Patterns of organismal abundance. On day 7, day 28, and day 42, the relative abundance of each genus was respectively compared to day 1. Those genera that were significantly changed among the groups are highlighted via red font. **b** Principle component analysis (PCA) based on the profile of functional genes, which supported the discrimination between the antibiotic group and the probiotic group at the functional level (PC1, 70.4%, *p* = 0.009; PC2, 12%, *p* = 0.6). However, neither the antibiotic group (PC1, *p* = 0.161; PC2, *p* = 0.229) nor the probiotic group (PC1, *p* = 0.123; PC2, *p* = 1) was distinguishable from the control group

To further probe how the distinct feed additives drive intestinal microbiota change, we compared the 16S gene-based profiles of bacterial phylogeny at the genus level at each of sampling times across the three groups. In total, eight genera were found changed significantly during the regimens by Kruskal Test (Additional file 6: Table S3). Among them, three abundant genera that include *Blautia*, *Roseburia*, and *SMB53* (representing 1.2, 0.9, and 0.8% of normal microbiota respectively) have changed significantly on day 7 (Fig. 4a). *Eubacterium*, *Roseburia*, *Clostridium*, *Clo_02d06*, *Tyzzerella*, and *Turicibacter* which respectively represent 0.2, 0.9, 14.0, 0.6, 0.2, and 0.5% of normal microbiota were significantly changed on day 28 (Fig. 4a); however, no genera were found significantly different in relative abundance across the three regimens on day 42 (Fig. 4a).

Further analysis revealed that, at day 7, there was no difference in microbiota beta diversity among the regimens (*F* = 2.08, *p* = 0.112). However, at day 28 such difference emerged (*F* = 4.72, *p* = 0.001) and then at day 42 it disappeared again (*F* = 0.58, *p* = 0.700). To test the functional distinction of microbiota at day 42, whole-metagenome sequencing of 10 fecal samples from each of the three groups at day 42 revealed a significant alteration of microbial functional profile among the three groups. Principle component analysis (PCA) based on KOs showed statistically significant discrimination between the antibiotic and the probiotic groups (PC1, 70.4%, *p* = 0.004; PC2, 12%, *p* = 0.563; Student’s *t* test; Fig. 4b), while neither the antibiotic group (PC1, *p* = 0.179; PC2, *p* = 0.115) nor the probiotic group (PC1, *p* = 0.111; PC2, *p* = 0.987) was distinguishable from the control group. Totally 1054 KOs were identified as functional markers associated with treatments (adjusted *p* < 0.1; Additional file 7: Table S4), which were then assigned to specific functional pathways (Additional file 8: Fig. S3; Additional file 9: Table S5A, B; “Methods”). Probiotics influenced as many pathways as antibiotics did; however, five pathways were altered only by antibiotics but not probiotics, including cell cycle–Caulobacter (ko04112), pentose and glucuronate interconversions (ko00040), synthesis and degradation of ketone bodies (ko00072), d-glutamine and d-glutamate metabolism (ko00471), and drug metabolism—other enzymes (ko00983). This might indicate disturbed energy metabolism and cell cycle under antibiotics. Thus, the distinct gut microbiota maturity rate between the antibiotic and the probiotic groups can lead to profound alteration of microbiota function.

### The key role of LP-8 in formation and development of bacterial correlation network in intestinal microbiota

**Fig. 5:**
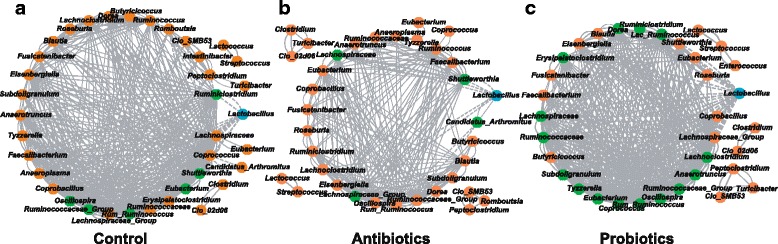
Bacterial co-occurrence network revealed the key role of *Lactobacillus* spp. in formation of bacterial correlation network in intestinal microbiota. Co-occurrence network of the control group (density = 0.329, centralization = 0.475; **a**), the antibiotic group (density = 0.213, centralization = 0.335; **b**) and the probiotic group (density = 0.355, centralization = 0.364; **c**) are shown. *Lactobacillus* spp. are highlighted as *blue circle*, while those genera directly correlated with *Lactobacillus* spp. are colored as *green*. The width of *gray lines* is proportional to Spearman correlation coefficient, while the type of lines represents the type of interaction (*solid line*: positive correlation; *dash line*: negative correlation)

To probe the potential mechanism underlying the distinct temporal patterns of gut microbiota maturation among the three regimens, we compared the corresponding co-occurrence networks among the bacterial genera. For each group, Spearman’s correlation coefficient was used to describe the adjacency relationship among genera. Intriguingly, in each of the three groups, the *Lactobacillus* spp. participated in the core interaction network (i.e., the largest sub-network; Fig. 5).

Compared to the control group (network density = 0.329; Fig. 5a), the inter-genera correlation in the antibiotic group was weaker (network density = 0.213; Fig. 5b), while in the probiotic group the correlation was stronger (network density = 0.355; Fig. 5c). On the other hand, the number of genera that were directly correlated with *Lactobacillus* spp. were very different. In the control groups, six genera were negatively correlated with *Lactobacillus* while this number was reduced to four in the antibiotic group but increased to 14 in the probiotic group. Furthermore, compared with the control group (*n* = 33), the number of genera participating in the core interaction network decreased in the antibiotic group (*n* = 27) and the probiotic group (*n* = 25). Interestingly, the inhibition of many intestinal non-*Lactobacillus* genera by the intestinal *Lactobacillus* spp. appeared to be the most prominent change taken place, suggesting enrichment of intestinal *Lactobacillus* spp. as induced by LP-8 feeding was one major driving force of the distinct global microbiota change in the probiotic group (Additional file 10: Fig. S4). Thus antibiotic feeding greatly disturbed and weakened the bacterial interacting network of the chicken gut microbiota while LP-8 feeding led to a strong interacting network where *Lactobacillus* spp. dominate. Consistently, the bacteriostasis effect by these enriched *Lactobacillus* spp. (due to administration of LP-8) against other intestinal bacterial genera might reduce nutrient consumption by the intestinal microbiota, which could have underlie the decline of FCR in the probiotic group (Fig. 1c).

**Fig. 6:**
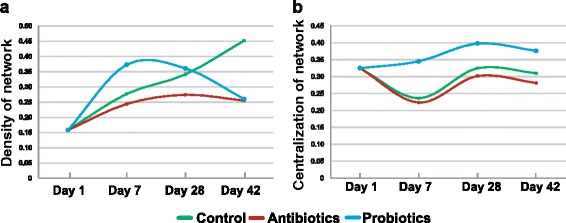
Temporal alteration of the density and centralization of bacterial correlation network under the three regimens. **a** Network density, which describes the portion of potential connections among bacteria. Before day 28, the density increased with time for all three groups and the probiotic group exhibit the highest density overall. Network density of the probiotic group peaked first and then dropped to the same level as the antibiotic group on day 42, while that of the control group gradually increased during the whole 42 days. **b** Network centralization, which measures the degree of dispersion of all node centrality scores in a network from the maximum centrality score obtained in the network. The highest centralization was found in the probiotic group (followed by the control group and the antibiotic group), which might suggest strongest resistance to propagation of pathogens in this group. The distinct impacts of antibiotics and probiotics on these key features of bacterial correlation network appeared to take place at an early phase

The distinct impacts of antibiotics and probiotics on bacterial correlation network appeared to take place at an early phase. In the first period (day 1 to 7), the correlations among genera in the antibiotic group have already been weakened as compared to the controls (network density of 0.244 and 0.277 respectively), whereas LP-8 feeding produced an opposite effect (network density of 0.373). In the next period (day 7 to day 28), such patterns were largely maintained, with the network density for control, antibiotics, and probiotics being 0.274, 0.341, and 0.361 respectively. Interestingly, in the final period (during day 28 to day 42), both antibiotics and LP-8 feeding reduced the mean correlation value of the networks, to 0.255 and 0.260 respectively (Fig. 6a). As for the centralization of network, the probiotic group always featured the highest concentrations of interacting genera during the whole trial, followed by the control and the antibiotic group (Fig. 6b). Thus both LP-8 and antibiotics, as feed additive, play a key role in formation and development of the web of bacterial interactions in broiler intestinal microbiota.

## Discussion

The growth-promoting effects of probiotics are dependent on specific probiotics used and the application level of probiotics [29]. Our data here suggested that LP-8 administration, just like antibiotic supplementation, significantly improved the growth-related matrices of broilers and also promoted immunological parameters in an industrial chicken farming setting. Moreover, LP-8 administration reduced FCR to a level equivalent with or lower than that under antibiotics, which is perhaps via inhibiting intestinal pathogens and thus reducing the nutrient consumption required for maintaining immunological activity [30]. Thus, probiotics as feed additive could bring economic benefits in the industrial farming of broilers.

Remarkably, using the control group as reference, we found that LP-8 accelerated the maturation of intestinal microbiota, whereas antibiotics delayed it. The consequence, at the late phase of the 42-day period, is the distinct microbial functions of the gut microbiota. This might potentially explain the growth-promoting effects of probiotics observed above, as a mature gut microbiota should be beneficial to the proper growth and development of host animals. Therefore, it is possible that the structural and functional dynamics of intestinal microbiota can be used as a signature to characterize, compare, and evaluate the feeding regimens in the poultry industry. One such example is IMMI, which can be used to quantitatively compare the speed of intestinal microbiota maturity under different feed additives or diets.

Notably, both antibiotic treatment and probiotic treatment exhibited positive effects on growth-related traits of the broilers; however, they had quite opposite effects on maturation of the intestinal microbiota: antibiotics delayed microbiota maturation, while probiotics accelerated the maturation. The concurrency of delayed microbiota maturation and improved growth in the antibiotic-feeding group appeared to contradict an important role of intestinal microbiota in growth. One explanation is that the observed enhancement in broiler growth in the antibiotic-feeding group is likely not a consequence of microbiota maturation but of the combination of three effects by the antibiotic-feeding regimen here (which was characterized by regular, feed-based administration of very low dose of antibiotics): (*i*) suppression of the growth of indigenous gut bacteria, which results in more nutrients for chicken for greater weight gain [31], (*ii*) inhibition of the colonization of those potentially harmful, non-indigenous bacteria in the intestine, which reduces gastrointestinal infections [32], and (*iii*) suppression of host immune response, e.g., by inducing anti-oxidative and anti-inflammatory activity of the host, which avoids biological damage caused by free radicals [33].

Finally, it was reported that probiotic *Lactobacillus* species might promote gut defense function by competitive exclusion of intestinal pathogens [34] or via activation and enhancement of local cell-mediated immunity against certain enteric pathogens [35]. However, it is not yet clear how LP-8 here specifically promoted the growth of the intestinal *Lactobacillus* spp. while also exhibited negative correlation with the 14 other bacterial genera, starting from the earliest phase of broiler development. Past studies have also observed that probiotic Lactobacillus strain feeding can greatly enhance the diversity of Lactobacilli in the ileum of broilers [36, 37]. It is possible that the introduced Lactobacillus strains such as LP-8 produce lactic acid and short-chain fatty acids in the chicken intestine, which reduce the intestinal pH value. The resulted more acidic environment (e.g., pH 4.5) prevents the growth of other intestinal bacteria such as Salmonella, *E. coli*, Campylobacter, and Clostridium, yet promotes the growth and diversity increase of *Lactobacillus* spp. in the chicken intestine [11, 38]. As the next step, we plan to test whether the beneficial effects of probiotics observed here are specific to particular probiotic strains, and to probe the molecular mechanisms underlying the impact of LP-8 introduction on intestinal microbiota. Nevertheless, our current study revealed remarkable difference in intestinal microbiota development between antibiotics and probiotics as broiler feed supplements. These findings support probiotics as an effective substitute for antibiotics as feed additive in the poultry industry, so as to reduce antibiotic residues from food animals and combat the spread of antibiotic resistance.

## Conclusions

Probiotic feeding induced the highest level of growth performance and immunity response. The maturation of intestinal microbiota was greatly accelerated by probiotic feeding, yet significantly retarded and eventually delayed by antibiotic feeding. Probiotic feeding might be an intestinal health-promoting attribute and may contribute to improved feed efficiency during the growth period. These findings support probiotics as an effective substitute for antibiotics as feed additive in the poultry industry, so as to reduce antibiotic residues from food animals and combat the spread of antibiotic resistance.

## Methods

### Study design

A total of 270 one-day-old Cobb 500 broilers were obtained from the Inner Mongolia Academy of Agriculture and then randomly divided into three groups. Each group included 90 chickens in six replicates (15 in each replicate). The control group was fed the base diet. The antibiotic group was fed the base diet plus the antibiotics, chlortetracycline and salinomycin, at 500 g/ton-of-feed each. The probiotic group was fed the base diet plus the probiotic strain *L. plantarum* strain IMAU10120 (LP-8) in the drinking water. The base diet, consisting mostly of corn and soybean meal, was provided by Inner Mongolia Guangye-Mufeng Biotechnology (Huhhot, China). LP-8 was originally isolated from traditional fermented dairy products of prairie herdsmen families in Inner Mongolia of China. The lyophilized P-8 was provided by Beijing Sci-plus Biotech (Beijing, China) and was added to the drinking water for chicken at a final concentration of 2 × 10^6^ CFU/ml.

Assuming a daily feed intake of ~100 g per bird, 2 × 10^6^ CFU LP-8 was selected as the per-feeding dose, at twice per day per bird, because the daily dose of probiotics for broiler was approximately 10^7^~10^8^ CFU/kg feed [39]. As it was not practical to enforce each bird to intake a defined dose of probiotics under the free eating mode, the probiotic feeding method was as below: at each probiotic feeding, bacterial freeze-dried powder that contained 2 × 10^6^ CFU of LP-8 was dissolved into 1 ml water and directly injected into the mouth of each broiler, once in the morning and once in the evening at each day [40, 41]. To ensure dose accuracy, the concentration of live bacteria in the powder was verified based on culture-based counting. Moreover, to verify the purity of the probiotic preparation, 70 clones were randomly picked from a culture plate derived from the bacterial freeze-dried powder. Genomic DNA was extracted from each of the clone and 16S genes amplified via universal bacterial 16S PCR primer (27F: AGAGTTTGATCCTGGCTCAG; 1492R: GGTTACCTTGTTACGACTT) and sequenced using ABI 3730. All of the 70 clones were confirmed as *Lactobacillus plantarum*, which validated the purity of our probiotic preparation.

This study was approved and carried out in accordance with the guidelines for the care and use of laboratory animals by the Inner Mongolia Agricultural University of China. Each of the six replicates in each group was housed in one pen, and a total of 18 pens were housed in the same room at the Key Laboratory of Dairy Biotechnology and Bioengineering, Education Ministry of China. Chickens were provided free access to feed and water during the 42-day trial.

Average daily gain (ADG) and average daily feed intake (ADFI) were monitored. All broiler chickens in each group were weighed individually at day 1, and then at each week during the full trial. The feed consumed for each pen was monitored on a weekly basis. ADG, ADFI, and feed conversion ratio (FCR; feed consumed/weight gain) were calculated for the periods of 1–21, 22–42, and 1–42 days.

### Detection of serum IgG and fecal SIgA and measurement of immune organ index

On day 14 and day 42 of the trial, two chickens from each replicate of each group were sacrificed by bleeding from the jugular vein, and 5 ml of blood was collected. Serum was prepared and stored at −20 °C until IgG was quantified. Approximately 1 cm of the jejunum was removed and quickly frozen in liquid nitrogen and then stored at −70 °C for RNA isolation. Peyer’s patches and another 1 cm of jejunum and cecal tonsils were removed and immediately fixed in 40% formaldehyde for immunohistochemistry analyses. Spleen and the remaining small intestine (approximately 10 cm from mid-duodenum to mid-ileum) and cecal tonsils were removed and washed with saline, and then placed in D-Hank’s solution (Beijing Huamaike Biotech, Beijing, China) at 4 °C for future use. Intestinal content (15 ml) was collected and mixed with an equal volume of phosphate buffered saline (PBS) (pH 7.14), and centrifuged at 800 g for 15 min. The supernatant was then stored at −20 °C until fecal SIgA was quantified. Serum total IgG and fecal SIgA were detected by enzyme-linked immunosorbent assay (ELISA). Their concentrations were then calculated from the standard curves.

On day 42, two chickens randomly selected from each replicate of each group were sacrificed. Thymus gland (on the right side) and bursal and spleen tissues were collected simultaneously and weighed for each chicken. The immune organ index (g/100 g) was calculated for each of the organs as WO/WB × 100, with WO being weight of the immune organ and WB weight of the chicken.

### Selection of time points for broiler fecal microbiota sampling

In broiler farming, there are three phases of chicken development from birth to slaughter: starter phase (day 1 to day 14), grower phase (day 15 to day 28), and finishing phase (day 29 to day 42) [42, 43]. The broilers are sent to a slaughter house at day 42 (i.e., tracking microbiota beyond day 42 is thus practically meaningless); therefore, the state of gut microbiota at day 42 was designated as “full maturity”. This does not rule out the possibility that under certain conditions, the microbiota can reach “full maturity” earlier (or later) than day 42. On the other hand, it was previously reported that composition of broiler ileum microbiota developed in a segmental manner, e.g., those at day 7~21 represent a relatively stable state while those at day 22~28 represent another relatively stable state [44, 45]. Therefore, we selected day 1, day 7, day 28, and day 42 as the four representative sampling time points that correspond to the various segments of microbiota development before slaughter.

### DNA extraction and qPCR

In each group, fecal samples were collected from the same 12 chicks on day 7, day 28, and day 42 respectively, and fecal samples of 10 randomly picked chickens were also collected on the first day of the trial. Fecal samples were collected aseptically and sample protectant added quickly before any further experiments. Fecal DNA was extracted from 0.4 g of fecal sample using a modified protocol of the QIAamp Stool Mini Kit (Qiagen, Germany). DNA was eluted in ddH_2_O and stored at −20 °C until use. The qPCR (primers listed in Additional file 2: Table S6) were performed using a Step-OneTM Real-Time PCR System (software version 2.2.2) (Applied Biosystems, USA). Reactions were performed in 96-well plates with SYBR® Premix Ex TaqTM II (Takara, Japan). All PCR were performed in triplicate with a reaction volume of 20 μl with 0.4 μM (final concentration) of each primer, a fixed amount of genomic DNA (100 ng), and an appropriate amount of ddH_2_O. The fluorescent product was detected at the last step of each cycle. Standard curves for each qPCR assay were generated by plotting the threshold cycle (CT) values against target copy numbers corresponding to serially diluted plasmid standards (Integrated DNA Technologies). The target copy numbers (*T*) were estimated by the equation: *T* = (*D*/(PL × 660)) × 6.022 × 1,023,133, where *D* (g/l) and PL (in base pairs) were the plasmid DNA concentration and length, respectively. Each standard curve was generated from at least five 10-fold plasmid dilutions in triplicate.

### Sequencing and analysis of the 16S amplicons

After DNA extraction from the broiler fecal samples, the V4-V5 region of the 16S rRNA genes were PCR-amplified with primers containing linker sequences (Forward: “GTACTCCTACGGGAGGCAGCA”; Reverse: “GTGGACTACHVGGGTWTCTAAT”) and sequenced on 454 GS FLX+. For quality filtering, sequences shorter than 400 bp or longer than 800 bp, as well as sequences containing fewer than two primer mismatches, uncorrectable barcodes, ambiguous bases, or homopolymer runs in excess of 8 bases, were removed using Parallel-QC [46] and QIIME [47]. Then the sequences were checked for chimeras using UCHIME [48] and assigned to operational taxonomic units (OTUs) using Parallel-META [49, 50] with a 97% threshold of pairwise identity, and then classified taxonomically using the Green genes reference database [51]. To standardize sequence counts across samples with uneven sampling, 2490 sequences were randomly selected per sample (rarefaction) and used as a basis to compare abundances of OTUs across samples.

Alpha diversity was calculated by (*i*) observed OTUs, (*ii*) Shannon Index, (*iii*) Simpson Index, and (*iv*) Chao1 index. Distance matrices (beta diversity) between the samples were generated on the basis of weighted Meta-Storms algorithms and reported according to principal coordinate analysis (PCoA). The Meta-Storms scoring function is a phylogeny-based algorithm that quantitatively evaluates the biological similarity/distance between microbiome samples on the OTU level, with high speed [52]. It performs bottom-up calculation by the traversal of 16S rRNA OTU phylogeny tree, considering both the OTUs’ relative abundances and the phylogenetic distances among OTUs, so that the beta diversity patterns of microbiome samples can be precisely revealed. Moreover, by normalizing for the copy number of 16S rRNA in each species, the Meta-Storms algorithm exhibits a better performance than results without copy number consideration. For statistical analysis including unsupervised clustering, PCoA, alpha and beta diversity, taxonomic distribution, and Wilcoxon Test, the results were generated using Parallel-Meta 3.3 [50].

### Modeling maturation process of gut microbiota using Random Forest algorithm

Random Forest regression, with a rarefied taxonomy table as input data, was used to regress relative abundances of taxa in the temporal profiles of gut microbiota of the controls against their chronologic age, using default parameters of the R implementation of the algorithm (R package “randomForest”, ntree = 5000, using default mtry of p/3 where *p* is the number of input 97%-identity taxa (features)). The Random Forest algorithm, due to its non-parametric assumptions, was applied to detect both linear and nonlinear relationships between taxa and chronologic age, thereby identifying those taxa that are highly correlated with age. In the control group, the regression consistently explained over 55.8% of the variance related to chronologic age. Ranked lists of taxa in the order of “feature importance” as reported by Random Forests were determined over 100 iterations of the algorithm. To estimate the minimal number of top ranking age-discriminatory taxa required for prediction, the “rfcv” function implemented in the “randomForest” package was applied over 100 iterations. The model was then applied to microbiota from the antibiotic group and the probiotic group. For the training of Random Forest model, a smoothing spline function was fitted between microbiota age and chronologic age of the host for the controls. “The microbiota age when the curve reaches the plateau” was defined as 100% maturity. The maturity index for each of the other two groups at a given time point was then calculated through the Random Forest model (using the control group as training dataset): the predict time point was used as *Y*-axis coordinate to represent the degree of maturity, while the actual time point as *X*-axis coordinate.

### Shotgun metagenome sequencing and analysis

Fecal samples at day 42 were sequenced by Illumina HiSeq 2500. Raw datasets of PE read files were analyzed via Parallel-QC (v1.0) to remove low-quality base pairs and sequence adapters using these parameters: Sliding window of 4:20, Minlength of 100, MinPhred of 25, and Percentage of MinPhred of 80. The paired-end and singleton reads were assembled using SPAdes v3.7.1. The open reading frames of the assembled scaffold sequence were annotated using MetaGeneMark (http://exon.biology.gatech.edu/GeneMark/). Bowtie2 (v2.2.1) aligner was used to map the reads to the assembled scaffold. KEGG Orthology was assigned through KAAS (http://www.genome.jp/tools/kaas/). Kruskal Test was used to identify the differential KOs among the three different groups by the R script in Parallel-META (version 3.3). Then the differentially enriched pathways were identified based on reporter score from the Z-scores of the individual KOs [53]. The Z-score for a KO is defined as below, where θ^−1^ is the inverse normal cumulative distribution, *P*_KO*i*_ is the adjusted *P* value for that KO:

$$ {Z}_{KO_i}={\theta}^{-1}\left(1-{P}_{KO_i}\right) $$

The aggregated Z-score for a KEGG pathway is below, where *k* is the number of KOs involved in the pathway:

$$ {Z}_{\mathrm{pathway}}=\frac{1}{\sqrt{k}}\sum {Z}_{KO_i} $$

Then the background distribution of Z_pathway_ was corrected by subtracting the mean (μ_*k*_) and dividing by the s.d. (*σ*_*k*_) of the aggregated Z-scores of 1000 sets of KOs chosen randomly from the whole metabolic KO network:

$$ {Z}_{\mathrm{adjustedpathway}}=\frac{Z_{\mathrm{pathway}}-{\mu}_k}{\sigma_k} $$

Z_adjustedpathway_ was then used as the final reporter score for evaluating the enrichment of specific pathways. A reporter score of ≥1.6 (90% confidence according to normal distribution) was set as a detection threshold for significantly differentiating pathways.

### Co-occurrence network analysis

The R package of “ccrepe” was used for calculating the Spearman’s correlation coefficient. Cytoscape 3.30 was used for network building. The R package of “corroplot” was used for generating the heat maps. In each of the three groups, 16S amplicon sequencing data on day 7, day 28, and day 42 were first pooled together to create the global network patterns, and then separately analyzed to illustrate the change of network density and network centralization.

### Availability of data and materials

Datasets supporting the conclusions of this article are available in the NCBI-SRA repository under Project Accession ID of PRJNA343678.

## Additional files

Additional file 1: Table S1. Effects of P-8 and antibiotics on Average Daily Gain (ADG), Average Daily Feed In (ADFI) and Feed Conversion Ratio (FCR) of broiler chicken. (DOCX 19 kb)

Additional file 2: Table S6. Sequences of the strain-specific primers used in RT-PCR of *Lactobacillus* spp. (DOCX 18 kb)

Additional file 3: Fig. S1. The absolute abundance of *Lactobacillus* spp. as determined by qPCR among the control, the antibiotics and the probiotics groups on day 7, day 28 and day 42. (PDF 1094 kb)

Additional file 4: Table S2. Pairwise Meta-Storms distances of all microbiome samples in this study for beta diversity analysis. (XLSX 137 kb)

Additional file 5: Fig. S2. Relative abundance of the 16 age-discriminating bacterial genera in the intestinal microbiota at each time point in the three broiler groups. (PDF 205 kb)

Additional file 6: Table S3. Comparison of relative abundance of all bacterial genera in broiler intestinal microbiota among the control, the antibiotics and the probiotics groups on day 7, day 28 and day 42. (XLSX 899 kb)

Additional file 7: Table S4. Comparison of microbial functional genes assigned to KEGG in broiler intestinal microbiota at day 42. (XLSX 197 kb)

Additional file 8: Fig. S3. KEGG metabolic pathways that differentiate the antibiotics group (or the probiotics group) from the control group. (PDF 2683 kb)

Additional file 9: Table S5. A. Enriched functional pathways in the antibiotic group as compared to the control group. B. Enriched functional pathways in the probiotics group as compared to the control group. (XLSX 11 kb)

Additional file 10: Fig. S4. Bacterial co-occurrence network of microbiota revealed a distinct inter-genera relationship driven by *Lactobacillus* spp. between the three regimens. (PDF 1485 kb)

## Abbreviations

ADFI: Average daily feed intake
ADG: Average daily gain
FCR: Feed conversion ratio
IgA: Immune globulin A
IgG: Immune globulin G
IMMI: Intestinal microbiota maturation index
PCA: Principle component analysis
SIgA: Secretory immune globulin A
