# Supplementary material for: Feed-additive probiotics accelerate yet antibiotics delay intestinal microbiota maturation in broiler chicken
Source: Microbiome. 2017 Aug 3;5:91. doi: 10.1186/s40168-017-0315-1 (PMC5541433; doi:10.1186/s40168-017-0315-1)
Supplement: Supplementary file 1 — Effects of P-8 and antibiotics on Average Daily Gain (ADG), Average Daily Feed In (ADFI) and Feed Conversion Ratio (FCR) of broiler chicken. (DOCX 19 kb) [file 40168_2017_315_MOESM1_ESM.docx]

**Additional file 1: Table S1. Effects of P-8 and antibiotics on Average Daily Gain (ADG), Average Daily Feed In (ADFI) and Feed Conversion Ratio (FCR) of broiler chicken**

| ADG (g/d) | Control | Antibiotics | Probiotics |
| --- | --- | --- | --- |
| Day 1-22 | 29.14±1.6 | 32.21±1.6 | 30.42±2.3 |
| Day 22-42 | 69.81±3.8 | 76.73±4.9 | 77.11±9.0 |
| Day 1-42 | 44.49±2.9 | 49.09±3.1 | 47.44±5.4 |

| ADFI (g/d) | Control | Antibiotics | Probiotics |
| --- | --- | --- | --- |
| Day 1-22 | 43.67±3.4 | 41.71±3.9 | 43.38±3.5 |
| Day 22-42 | 130.52±3.4 | 135.56±3.3 | 126.92±10.0 |
| Day 1-42 | 79.52±1.5 | 82.31±2.7 | 77.79±4.9 |

| FCR | Control | Antibiotics | Probiotics |
| --- | --- | --- | --- |
| Day 1-22 | 1.50±0.1 | 1.30±0.1 | 1.42±0.1 |
| Day 22-42 | 1.87±0.1 | 1.78±0.1 | 1.67±0.2 |
| Day 1-42 | 1.79±0.1 | 1.68±0.1 | 1.66±0.2 |
