# Supplementary material for: Feed-additive probiotics accelerate yet antibiotics delay intestinal microbiota maturation in broiler chicken
Source: Microbiome. 2017 Aug 3;5:91. doi: 10.1186/s40168-017-0315-1 (PMC5541433; doi:10.1186/s40168-017-0315-1)
Supplement: Supplementary file 2 — Sequences of the strain-specific primers used in RT-PCR of Lactobacillus spp. (DOCX 18 kb) [file 40168_2017_315_MOESM2_ESM.docx]

**Additional file 2: Table S6. Sequences of the primers used in RT-PCR of *Lactobacillus* spp.**

| **Target microbial groups** | **Primer sequences** | **Target gene** |
| --- | --- | --- |
| *Lactobacillus* | F:AGCAGTAGGGAATCTTCCA | 16S rRNA gene |
|  | R:CACCGCTACACATGGAG |  |
| *L. plantarum* | F:TGGATCACCTCCTTTCTAAGGAAT | 16S rRNA gene |
|  | R:TGTTCTCGGTTTCATTATGAAAAAATA |  |
| *L. salivarius* | F:TAAACAAAGTATTCGATAAATGTAC | 16S rRNA gene |
|  | R:GTCGTAACAAGGTAGCCGTAGGA |  |
| *L. gasseri* | F:TGCTATCGCTTCAAGTGCTT | 16S rRNA gene |
|  | R:AGCGACCGAGAAGAGAGAGA |  |
| *L. ruminis* | F:CACCGAATGCTTGCAYTCACC | 16S rRNA gene |
|  | R:GCCGCGGGTCCATCCAAAA |  |
| *L.brevis* | F:ATTTTGTTTGAAAGGTGGCTTCGG | 16S rRNA gene |
|  | R:ACCCTTGAACAGTTACTCTCAAAGG |  |
| *L. sakei* | F:GATAAGCGTGAGGTCGATGGTT | 16S rRNA gene |
|  | R:GAGCTAATCCCCCATAATGAAACTAT |  |
| *L.acidophilus* | F:GAAAGAGCCCAAACCAAGTGATT | 16S rRNA gene |
|  | R:CTTCCCAGATAATTCAACTATCGCTTA |  |
| *L. reuteri* | F:ACCGAGAACACCGCGTTATTT | 16S rRNA gene |
|  | R:CATAACTTAACCTAAACAATCAAAGATTGTCT |  |
| *L. paracasei* | F:ACATCAGTGTATTGCTTGTCAGTGAATAC | 16S rRNA gene |
|  | R:CCTGCGGGTACTGAGATGTTTC |  |
| *L. casei* | F:CTATAAGTAAGCTTTGATCCGGAGATTT | 16S rRNA gene |
|  | R:CTTCCTGCGGGTACTGAGATGT |  |
