# Supplementary figures and images for: Feed-additive probiotics accelerate yet antibiotics delay intestinal microbiota maturation in broiler chicken
Source: Microbiome. 2017 Aug 3;5:91. doi: 10.1186/s40168-017-0315-1 (PMC5541433; doi:10.1186/s40168-017-0315-1)

Figure S1

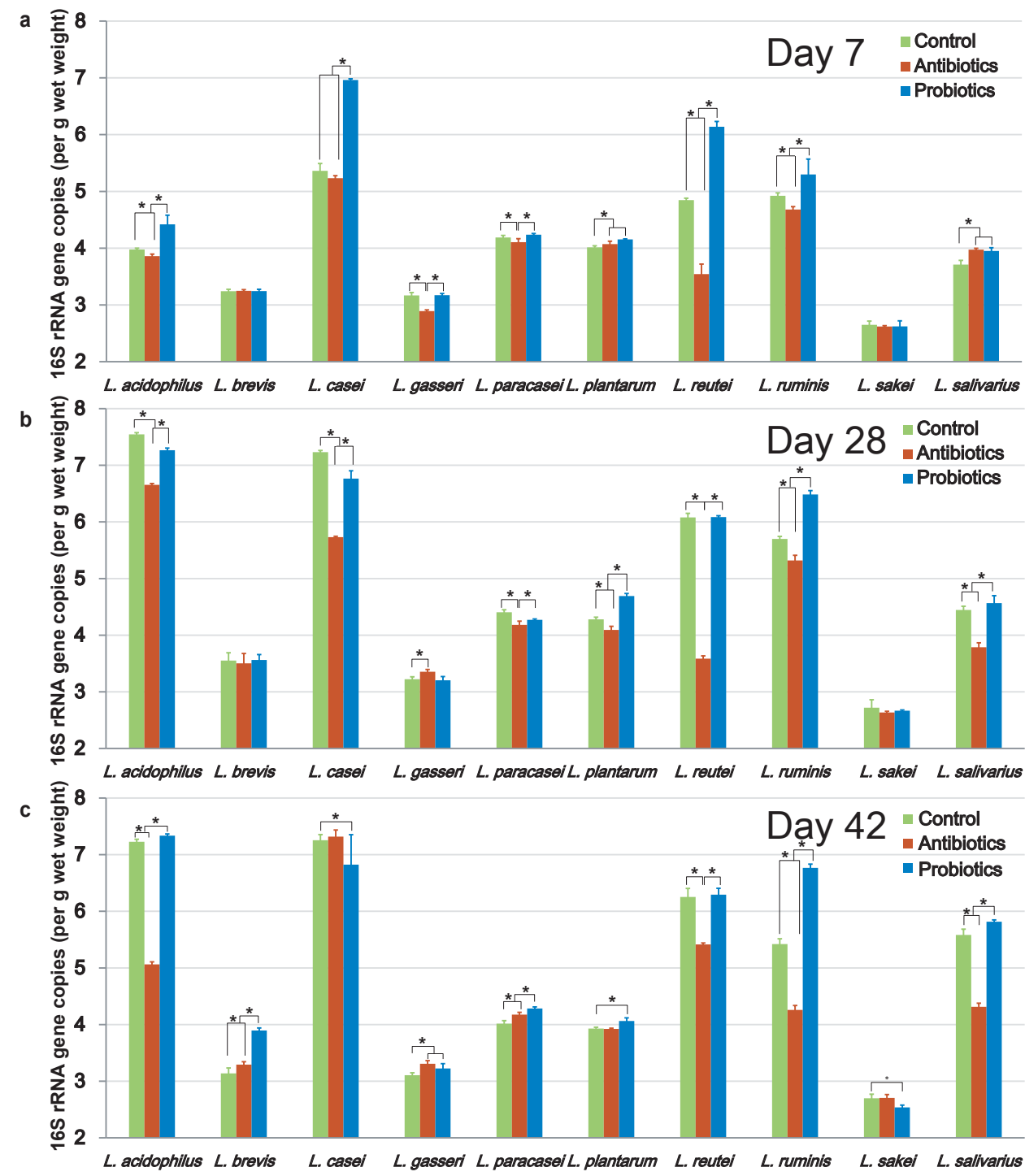

Supplement: Supplementary file 3 — The absolute abundance of Lactobacillus spp. as determined by qPCR among the control, the antibiotics and the probiotics groups on day 7, day 28 and day 42. (PDF 1094 kb) [file 40168_2017_315_MOESM3_ESM.pdf]

## Figure S2

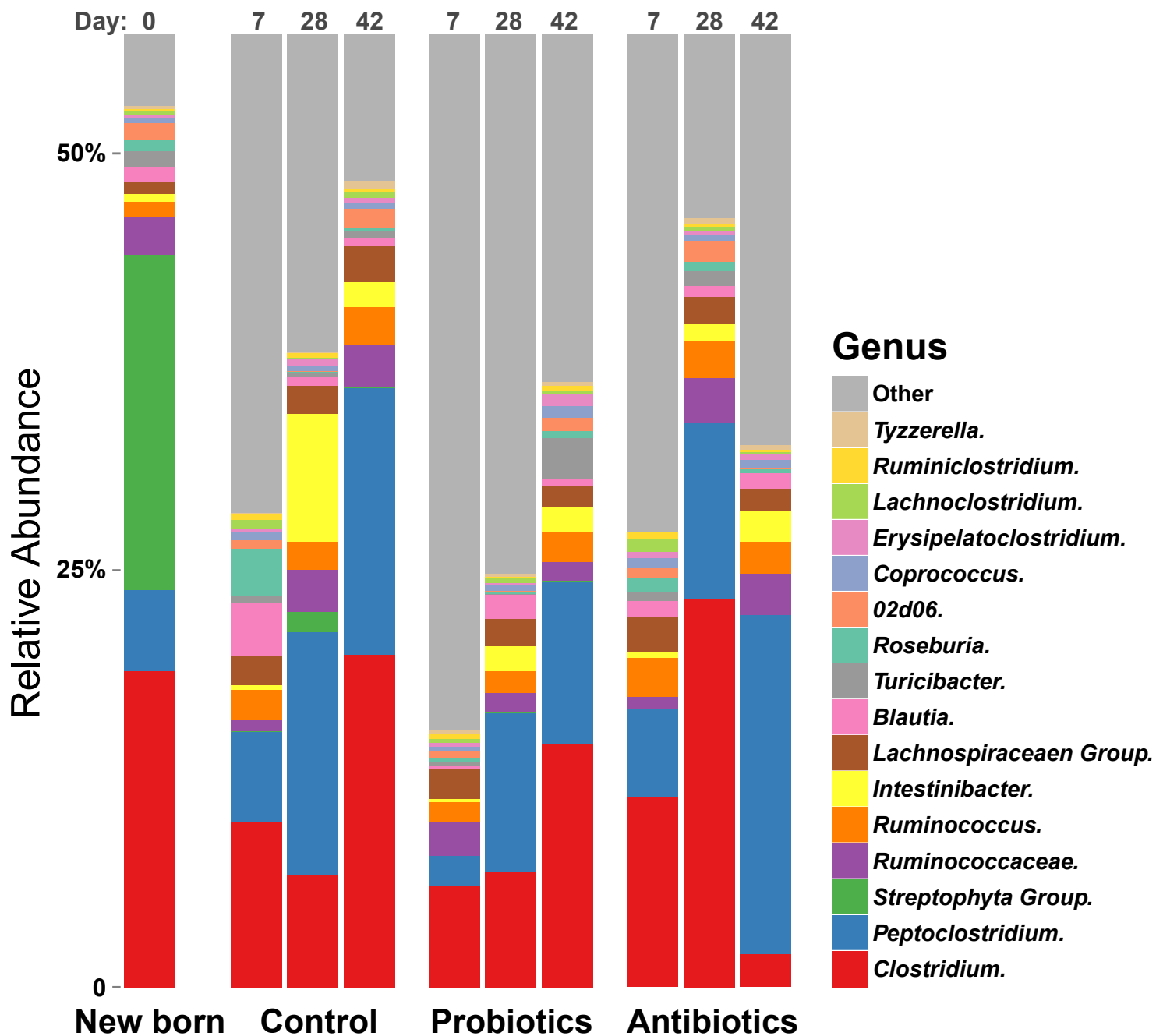

Supplement: Supplementary file 5 — Relative abundance of the 16 age-discriminating bacterial genera in the intestinal microbiota at each time point in the three broiler groups. (PDF 205 kb) [file 40168_2017_315_MOESM5_ESM.pdf]

**Figure S3**

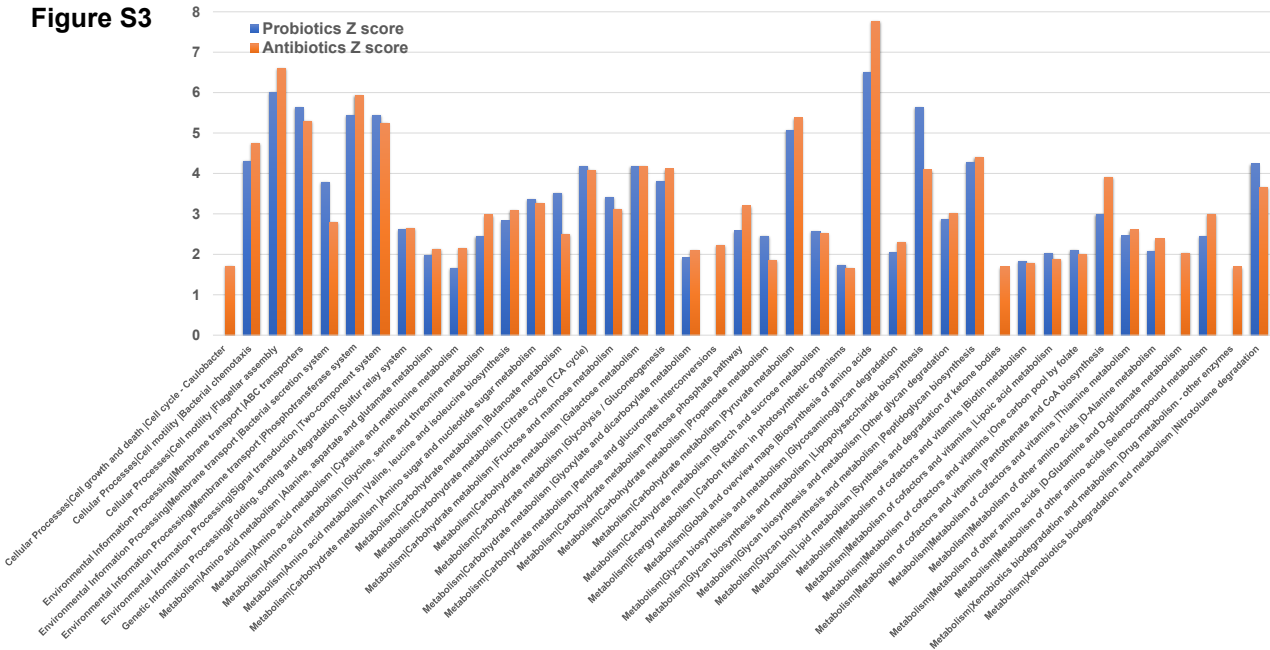

Supplement: Supplementary file 8 — KEGG metabolic pathways that differentiate the antibiotics group (or the probiotics group) from the control group. (PDF 2683 kb) [file 40168_2017_315_MOESM8_ESM.pdf]

# Figure S4

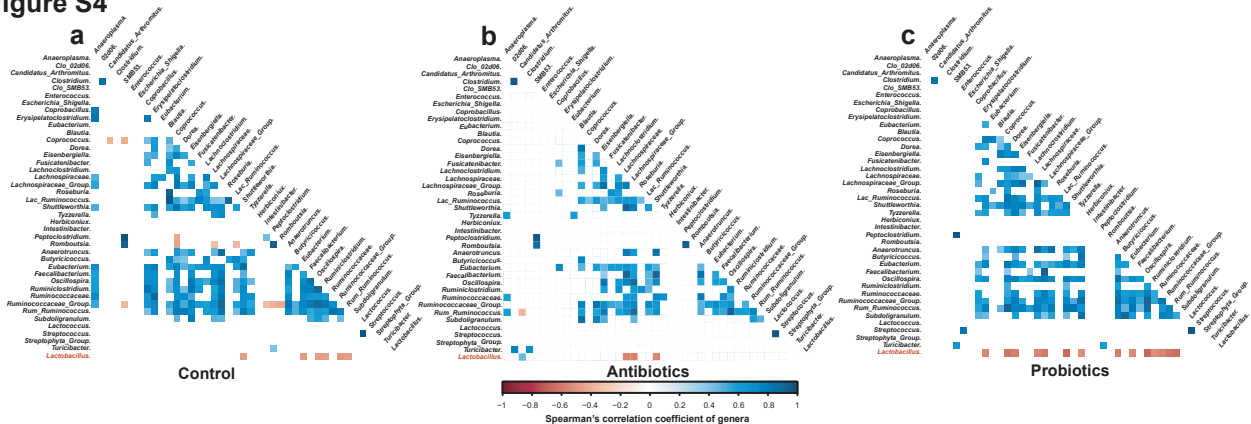

Supplement: Supplementary file 10 — Bacterial co-occurrence network of microbiota revealed a distinct inter-genera relationship driven by Lactobacillus spp. between the three regimens. (PDF 1485 kb) [file 40168_2017_315_MOESM10_ESM.pdf]
